# Neuroendocrine carcinoma of the cervix presenting as intractable hyponatremic seizures due to paraneoplastic SIADH—a rare case report and brief review of the literature

**DOI:** 10.3332/ecancer.2014.450

**Published:** 2014-07-31

**Authors:** Santhosh Kuriakose, N Umadevi, Sheela Mathew, NK Supriya, KP Aravindan, DS Smitha, G Amritha Malini

**Affiliations:** Government Medical College, Medical College P.O., Kozhikode, Kerala 673001, India

**Keywords:** neuroendocrine carcinoma (NEC), paraneoplastic syndromes, SIADH

## Abstract

Herein is presented an interesting case of small-cell neuroendocrine carcinoma of the cervix which initially manifests as seizures due to hyponatremia caused by paraneoplastic syndrome of inappropriate anti diuretic hormone (SIADH). Awareness of a paraneoplastic syndrome at presentation can lead to early diagnosis and early initiation of treatment. The management is also unique in that it combines treating the paraneoplastic aspects as well as targeting the tumour itself. Multimodality treatment gives the best outcome in this aggressive tumour.

## Introduction

Endocrine paraneoplastic syndromes associated with gynaecological malignancies are rare. Small-cell carcinoma of the cervix is known to be a rare and aggressive tumour. We present an interesting case of a neuroendocrine carcinoma of the cervix that manifests as seizures associated with hyponatremia. It was found to be a neurologic manifestation of paraneoplastic syndrome of inappropriate anti diuretic hormone (SIADH). Paraneoplastic syndrome in the form of SIADH manifested even before a cancer diagnosis could be made. Awareness of the atypical presentation of this aggressive neoplasm can lead to an early diagnosis and treatment. Combining multiple treatment modalities like radical surgery and concurrent chemoradiation gives reasonable outcomes in these aggressive tumours.

## Case presentation

A 50-year-old multiparous postmenopausal lady was admitted with complaints of vomiting and adult-onset seizure associated with altered sensorium. There had been another episode of seizure one month prior. Clinically, she had Eastern Cooperative Oncology Group (ECOG) performance status of 2. A neurological examination was normal except for drowsiness. She was evaluated by a physician. Clinically, there was no evidence of any systemic disease. She was euvolemic with normal hydration and normal urinary output. Liver and renal parameters were within normal limits. Evaluation revealed hyponatremia (serum Na 116 mEq/L). A computed tomography (CT) scan of the brain and chest x-rays were normal. A basic metabolic panel revealed sodium 116 mmol/L, chloride 89 mmol/L, potassium 3.6 mmol/L, magnesium 2.3 mmol/L, calcium 8.1 mg/dL, phosphorus 3.8 mg/dL, blood sugar 118 mg/dL, and plasma osmolality (P_Osm_) 242 mOsm/kg. Urine sodium, osmolality, and specific gravity were found to be raised, the values being 70 mmol/L, 360 mOsm/kg, and >1.050, respectively. In view of the hyponatremia associated with neurological features, plasma hypo-osmolality, increased urinary sodium, and hyperosmolar urine, SIADH was diagnosed.

SIADH is always a diagnosis of exclusion. Other causes of hyponatremia (adrenal and thyroid insufficiency, physiological sources of vasopressin stimulation such as CNS lesions, pulmonary disease) were ruled out. An MRI (magnetic resonance imaging) scan revealed an empty sella syndrome. Serum cortisol level was 26.54 μg/dL (normal 5–25 μg/dl). ACTH (adrenocorticotrophic hormone) stimulation test done revealed serum cortisol level >63.44 μg/dL, and pituitary insufficiency was ruled out. Thyroid function tests were found to be within normal limits [T3–94 ng/dL (84–201 ng/dL), T4–7.5 (5.5–11.7 μg/dL), TSH–1.99 mIU/L (0.5–5.5 mIU/L]. In view of the normal pituitary, adrenal, and thyroid functions, the empty sella was taken as an incidental finding on MRI scan.

**Figure 1.**
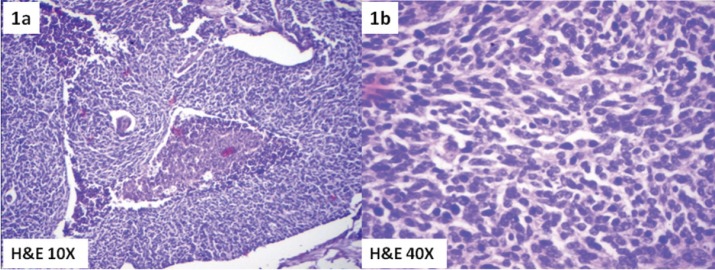
Photomicrograph showing small-cell neuroendocrine carcinoma of cervix. (1a) H& E 10X and (1b) H&E 40X.

Meanwhile, she gave a history of postmenopausal spotting per vaginam and hence was evaluated by a gynecologist. Abdominal examination revealed a well-defined cystic rounded mass of 15 x 8 cm, palpable in the hypogastrium. On pelvic examination there was a 3 x 3 cm hard exophytic growth arising from the anterior lip of the cervix. A vaginal nodule 0.5 x 0.5 cm noncontiguous with the cervical tumour was found near the posterior fornix. The uterus was normal size, and a right adnexal cystic mass of 15 x 8 cm was also identified. Bilateral parametrium were free. A biopsy of the cervix revealed a small-cell neuroendocrine carcinoma of the cervix (Figure 1a & 1b) which explained the hyponatremia and neurological findings as of the paraneoplastic manifestation due to ectopic hormonal production by the tumour.

**Figure 2.**
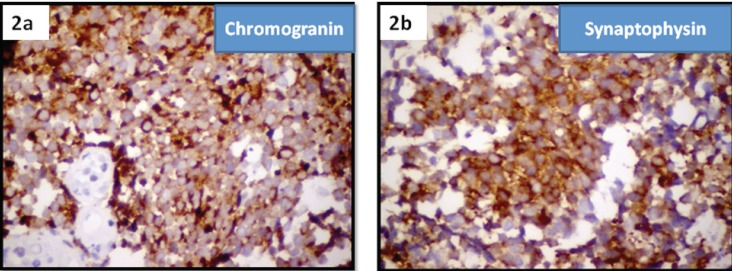
Immuno histochemistry of small-cell neuroendocrine carcinoma cervix showing positivity for (2a) chromogranin and (2b) synaptophysin.

Immunohistochemistry confirmed the diagnosis by staining positive for synaptophysin and chromogranin markers of neuroendocrine tumours (Figure 2a and 2b).

**Figure 3.**
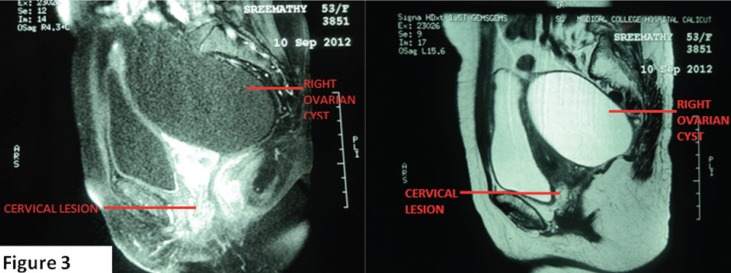
MRI pelvis showing well-defined iso-hyperintense lesion in the cervix (2.8 x 2.4 cm) and a large simple right ovarian cyst hypointense on T1Wand hyperintense on T2W.

Tumour markers CA 125 and CEA (carcinoembryonic antigen) were found to be within normal limits. A magnetic resonance imaging (MRI) scan of the abdomen and pelvis revealed a well-defined iso-hyperintense lesion, 2.8 x 2.4 cm in the cervix, and a large well-defined cystic lesion 12 x 9.5 cm in size hypointense on T1W (T1 weighted image) and hyperintense on T2W (T2 weighted image) extending from the right lower abdomen into the pelvis posterolateral to the uterus, with no solid components/septations (Figure 3).

**Figure 4.**
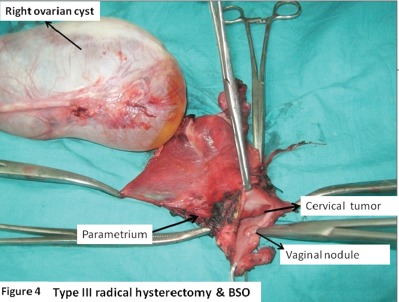
Type III radical hysterectomy and BSO showing right ovarian simple cyst (15 x 8cm); cervical tumour 3.5 x 3.5 cm, mainly involving the endocervix; and posterior vaginal flap tumour nodule.

An ultrasound with a Doppler of the abdomen was suggestive of a benign ovarian cyst. A bone scan was found to be normal. A diagnosis of small-cell neuroendocrine carcinoma cervix FIGO (International federation of obstetrics and gynaecology) stage IB1 associated with SIADH was made. The ovarian cyst was provisionally taken to be a benign cyst. The patient was treated with a type III radical hysterectomy and BSO (bilateral salphingoopherectomy) with pelvic lymphadenectomy. Intraoperatively, there was a right ovarian simple cyst 15 x 8 cm. The cervix revealed an exophytic growth 3.5 x 3.5 cm, mainly involving the endocervix. The posterior vaginal wall revealed a nodule of 0.5 x 0.5 cm. Also, a left parametrial nodule of 0.5 x 0.5 cm was identified which was removed in the specimen (Figure 4).

Histopathology revealed small-cell neuroendocrine carinoma-extending to more than one-half of fibromuscular cervix. Tumour emboli were seen in the left parametrium and vaginal flap. All margins were free of tumour. The right ovarian tumour was a benign serous cystadenoma. All lymph nodes (21 in number) were found to be uninvolved. Hyponatremia was spontaneously corrected in the postoperative period (serum sodium 136 mmol/L). Sequential chemotherapy and radiotherapy were given as adjuvant treatment. The sequence of treatment was three cycles of chemotherapy consisting of cisplatin and etoposide, followed by radiotherapy, which was then followed by three more cycles of the same chemotherapy. The chemotherapy schedule consisted of each cycle of etoposide 100 mg/m^2^ for three days and cisplatin 75 mg/m^2^ in divided doses with hydration, given three weeks apart. A radiotherapy regimen followed for carcinoma of the cervix was given.

Although the patient remained disease-free locoregionally, it recurred a year later as secondaries in the liver and brain. The patient succumbed to the disease one month later.

## Discussion and review of the literature

Tumour secretion of antidiuretic hormone (ADH), also known as arginine vasopressin (AVP), and atrial natriuretic peptide (ANP), results in SIADH, which is characterised by euvolemic hyponatremia and affects 1% to 2% of all cancer patients. ADH causes excessive water resorption in the collecting ducts. This increased intravascular volume leads to increased renal perfusion along with a substantial decrease in proximal tubular absorption of sodium. ANP binds to a specific set of receptors, resulting in increased renal sodium excretion [1]. These patients do not become hypervolemic because of the natriuretic mechanisms that are activated. This restores euvolemia, but worsens serum sodium levels. Together, these mechanisms cause euvolemia and dilutional hyponatremia. Small-cell carcinoma of the cervix, otherwise called neuroendocrine small-cell carcinoma, accounts for up to 2% of cervical carcinomas [1–4]. They carry a worse prognosis compared to poorly differentiated squamous cell carcinoma of the cervix [5, 6]. With the prevalent use of immunohistochemistry in pathology, more of these cases are being diagnosed accurately [7, 8]. The median age of diagnosis is in 55 years (range: 21–87 years). The usual presenting symptom is vaginal bleeding, and a clinically detectable cervical mass is present in most cases. Rarely, abnormal PAP smears have led to the diagnosis [9]. Clinical presentation with features of hyponatremia secondary to ectopic hormone production is extremely rare. Being an adenocarcinoma, its location is more likely to be in the endocervical canal and hence will require endocervical curetting in-addition to biopsing the cervix for obtaining an adequate specimen for pathology. Pathologic features of aggressive tumours have high mitotic rate, extensive necrosis, frequent lymphvascular space invasion (LVSI). They are frequently seen in strong association with HPV (human papilloma virus) 18, as has been noted [10–11]. Immunohistochemical analysis reveal the presence of neuroendocrine hormones and polypeptide hormones within the cells, and this can differentiate from other morphologically similar entities like basaloid squamous cell carcinoma, embryonal rhabdomyosarcoma, lymphoma, and undifferentiated carcinoma arising from the lower uterine segment. In addition, one must differentiate small-cell neuroendocrine carcinomas from poorly differentiated squamous cell carcinoma (SCC) with neuroendocrine features. Even though NECs (neuroendocrine carcinomas) of the cervix are staged as any other cervical cancer [12], they do not follow the locoregional pattern of spread of cervical cancer. Even in Stage IB1 tumours, there is 40% pelvic lymph node involvement and 60% LVSI, and this correlates with its aggressiveness and poor prognosis [4, 13, 14]. The final histopathology in this case also revealed poor prognostic features, such as the tumour extending to more than one-half of the cervical stroma and tumour emboli in the left parametria and vaginal flap. A metastatic workup including CT or PET/CT is highly recommended [15, 16]. The mean time for recurrence was 19.9 months. Our patient had the recurrence one year later in the liver and brain. The bones, supraclavicular lymph nodes, and lungs are the most common sites of metastasis.

Although this case is most interesting from a clinical point of view, it is even more significant because it calls attention to the potential endocrine activity of such small-cell carcinomas of the cervix. Because of the lack of clinical endocrine syndromes in most patients with small-cell carcinoma of the cervix, many have suggested that the hormonal polypeptides in these tumours are secreted in an inactive form or in an insufficient amounts to produce clinically detectable syndromes or may be are rapidly inactivated in the circulation [17, 18]. Hyponatremia in cancer patients occurs due to salt depletion and SIADH due to ectopic hormone production (AVP and ANP). It is defined as a serum sodium lower than 130 mEq/L, and has a reported incidence of 3.7%. Mild hyponatremia causes nonspecific symptoms, but rapid onset with value below 115mg/dL results in serious neurological deficits (psychotic behavior, abnormal reflexes, papilledema, focal neurologic signs, seizures, coma, and respiratory arrest) [19, 20]. Drugs reported to cause SIADH include cyclophosphamide, vinca alkaloids, as well as carboplatin, and cisplatin.

The optimal therapy for paraneoplastic SIADH is treating the underlying tumour, which if successful, can normalise the sodium level in a matter of weeks [20]. In our case, the spontaneous correction of serum sodium from the preoperative 116 mmol/L to the postoperative value of 136 mmol/l profoundly speaks of the endocrine activity of the tumour that had resulted in hyponatremia and the neurological manifestations.

## Management

Neuroendocrine carcinoma with SIADH is a unique entity and makes this case unique. The treatment is mainly based on the stage of the disease. In early stage (FIGO Stage IA, IB1, and IIA), surgery is the mainstay of management and is shown to have improved outcomes [21–30] compared to radiotherapy. Furthermore, the excision of the hormone-producing tumour will result in the correction of electrolyte abnormalities. The case presented proves this point well. As multimodality treatment is known to improve a patient’s outcome, we decided to give radiotherapy with concurrent chemotherapy as adjuvant treatment. We gave due consideration for the poor prognostic factors, namely, cervical stromal invasion of more than 50% and tumour emboli in the parametria and vagina. A combination chemotherapy using cisplatin and etoposide is the most effective and commonly used treatment modality in NEC [31–33]. Adjuvant radiation following surgery in early stage disease is not supported by retrospective data [23, 35], but many studies show improved outcomes with multi-modality regimen in early stage of the disease [36, 26].

Chemoradiation with etoposide/cisplatin (EP) has been used in the treatment of stage IA–IVB disease [36]. The exacerbation of SIADH with the use of cisplatin does not seem to be addressed in any of these studies as the clinically evident SIADH is, in fact, sparsely reported. It may be noted that fluid restriction, which is suggested in the treatment of SIADH, cannot be carried out in view of the renal toxicity of the agent. Hence it may be prudent to give sequential treatment with radiation followed by chemotherapy as it may result in shrinkage of the tumour thereby achieving control over SIADH before initiating chemotherapy.

Early recurrence in the liver and brain is evidence of the aggressive nature of the tumour. Distant sites of recurrence are more common (28%) than local failure (13%) [36]. Our patient was locoregionally-free of disease at the time when she developed distant metastasis. Compared to limited-stage small-cell lung cancer, small-cell cervical cancers seems to have a better five-year survival rate, 20% versus 36% [25, 33]. Clinical stage was the only independent predictor for disease-free survival, 80% at three years for stage I/II, and 38% for stage III/IV [36].

For advanced stage disease, the treatment is largely palliative. Vincristine/doxorubicin/cyclophosphamide and topotecan are considered as alternate or second-line therapies extrapolating from small-cell lung cancer [37, 38].

The management algorithm proposed by Chan [21] and its modification [39] may be helpful in treating NEC of the cervix. The gist of the treatment is that in tumour less than 4 cm radical hysterectomy with lymphadenectomy followed by etoposide/platinum chemotherapy, and tumours larger than 4 cm with disease confined to pelvis, neoadjuvant chemotherapy with cisplatin and etoposide followed by locoregional treatment (radical surgery/ radiotherapy) may give the best results.

## Conclusion

We described a rare case of small-cell neuroendocrine carcinoma of cervix that initially manifested as a neurological disorder due to paraneoplastic SIADH. This case explains the methodological evaluation of euvolemic hyponatremia and its neurological manifestations, the judicious use of investigations, and its interpretation, the relevance of biopsy and immunohistochemistry, and use of multimodality management in the treatment of this complex disorder in the light of evidence found in the available literature. It is important to recognise such a paraneoplastic presentation at an early stage, so that treatment can be initiated sufficiently early, as treatment during the later stages is known to be invariably ineffective.
